# “Our riches are our family”, the changing family dynamics & social capital for new migrant families in Australia

**DOI:** 10.1371/journal.pone.0209421

**Published:** 2018-12-26

**Authors:** Nidhi Wali, Andre M. N. Renzaho

**Affiliations:** 1 Humanitarian and Development Research Initiative, School of Social Sciences and Psychology, Western Sydney University, Locked Bag 1797, Penrith, New South Wales, Australia; 2 School of Social Sciences and Psychology, Western Sydney University, Locked Bag 1797, Penrith, New South Wales, Australia; Middlesex University, UNITED KINGDOM

## Abstract

Immigration from collectivist cultures to Western countries often results in loss of social capital and changing family dynamics leading to isolation and acculturative stress. This study explored the impact of social and cultural changes experienced by seven migrant communities residing in Greater Western Sydney, Australia. It deconstructed the role of local community and networks in their initial settlement in absence of traditional forms of community support. Data were collected through fourteen focus group discussions (164 participants). Five major themes emerged: (i) changing gender roles and women empowerment; (ii) sending money home; (iii) culture shock and increased intercultural conflict; (iv) change in lifestyle from collective to individual culture; and (v) role of extended community in mitigating culture shock. These findings suggest that community interventions aimed at improving cultural and social engagement of migrants employ social capital framework. This will ensure enhanced communication within migrant families and communities from different cultural and linguistic backgrounds.

## Introduction

Migration is a complex phenomenon and is associated with a range of challenges of adapting and settling in the new environment and culture [1–3]. Over the past few decades transnational migration from non-Western and non-English speaking societies to industrialised Western societies of the United States, Australia, Canada and Europe has become increasingly common [4–6]. For example, in 2015 the number of persons living outside their country of birth voluntarily for economic reasons or because of conflicts was estimated at 244 million (or 3.3 percent of the world's population) worldwide and more than two thirds of all international migrants were found to live in high-income countries. [7]

Australia is one of the high-income developed countries that accepts a significant number of migrants. The 2011 census data show that 28.2 percent of the Australian population were born overseas and 67 percent of recent arrivals speak a language other than English at home [8, 9]. Although being a multicultural society that embraces plural cultures and ethnic diversity, Australia’s mainstream culture is individualistic in nature with a modern welfare system [4, 10]. This system facilitates migrants’ easy access to education, English language courses, employment opportunities, and health services [2]. It associates the initial process of integration and settlement with finding employment, learning the local language and familiarising oneself with the new system and culture, compounded by challenges such as financial constraints, racism and discrimination [2, 11].

Prior to migration, migrants from non-Western and non-English speaking backgrounds mostly come from cultures that value collectivism, characterised by interdependence, and harmonious blending within a community and family [2, 12]. Collectivism is represented by ‘individuals who are interdependent within their in-group (family, tribe, and nation) and regard the goals of the in-group above their personal goals’ [13]. Within collectivistic cultures people identify themselves with their family, extended relatives and friends and community [13, 14]; the perspective of family extends beyond the immediate family to include close uncles, aunties, cousin brothers sisters and relatives [12]; and there are defined hierarchical role divisions within families. Post-migration these cultural values are replaced with individualistic culture valuing individuation and autonomy [13]. However, the role of individualism as compared to collectivism in the formation of culture, beliefs or values will differ within and between migrant groups depending on their pre-migration experiences, level of education, length of stay in Australia, migration paths and other characteristics, such as gender and age. [3]

Upon migration, migrants experience challenges of establishing social identity and networks, finding employment, negotiating the new system, learning English and looking after their own health and well-being [2, 3, 15]. In addition, migrants experience role reversals in families between genders, and parents and their children, intergenerational acculturative gap leading to conflict and poor communication within families [2, 3, 15]. These experiences along with absence of close family and social networks can further negatively affect their ability to cope, leading to acculturative stress.

Acculturative stress refers to poor psychological, physical and social well-being of migrants that result from the process of acculturation or the process of negotiating between cultures [16]. The acculturative stress increases as migrants struggle to adjust and adapt to their new cultural environment, especially negotiating differences between the original culture and host culture in relation to cultural norms and values, social customs, political landscape, and education standards. The effect of acculturative stress on health and well-being of migrants may vary according to their pre-migration experiences, migration status, socio-economic status, and proficiency in the host language [17, 18]. Having social capital can mitigate the negative effects of acculturation and contribute positively to migrants’ integration and settlement experiences [19]. Social capital refers to "networks with shared norms, values and understanding which facilitate co-operation within or among groups" [20]. It is characterised by two critical dimensions, bonding and bridging. Bonding social capital refers to links to people based on a sense of common identity, people who share similar culture and ethnicity, such as families and close friends while bridging social capital refers to relations between ethnic groups as well as between immigrants and the native-born. [4]

New migrants and migrant families, staying less than ten years in the host country, might not have sufficient access to their own extended family members, relatives, friends and even immediate family members in some cases. Their new families extend through time and geography to include local people with similar background, ethnicity and culture [21]. Higher levels of social capital are often associated with many desirable outcomes, such as faster social and economic development, greater effectiveness of political systems, and better health [22, 23]. Research has shown that social capital has a positive influence on the acquisition and accumulation of human and financial capital. This acquisition increases migrant participation in various social and financial initiatives, hence facilitating a positive settlement experience [22, 24].

The present study attempts to employ the social capital theory to examine the ability of migrants to secure benefits from the social membership and networks they establish [23] and its influence on their settlement and integration journey after arriving to Australia. Through the social capital lens, the aim of the study was threefold: 1) to understand the impact of social and cultural changes on migrants settlement trajectory; 2) to explore how migrants cope with changing dynamics within the family unit and the loss of traditional forms of community support; and, 3) to examine the role of social capital in the settlement experiences of migrants and their families.

## Methods

### Study design and governance

The research was carried out in Greater Western Sydney (GWS). Greater Western Sydney is among the most multicultural regions of the metropolitan area of Sydney, Australia, with 28 percent of its population born overseas [9]. This research was an exploratory qualitative design targeting the largest emerging migrant groups in the region, namely migrants of Afghanistan, Sub-Saharan African countries (including Eritrea, Ghana, Liberia, Rwanda, Sierra Leone, Sudan and Uganda), Bangladesh, Burma, Nepal and India. The study included migrants from all migration streams, including refugees and humanitarian entrants, family reunion, and skilled migration. A Migrant Review Panel (MRP) was established as a de facto community advisory committee comprising community leaders, academicians, representatives from community organisations, and lay representatives from migrant communities. The MRP members also contributed to all aspects of the project including participant recruitment, development of the interview guide, feedback on the study findings, and feeding the results back to the community. Involving the MRP at each stage brought guidance and insight and ensured rigour and trustworthiness of the research methods as it established credibility. The study was approved by the Western Sydney University Human Research Ethics Committee (H11213).

### Recruitment and data collection

The study procedures have been described elsewhere [15, 25]. Briefly, community mobilisation and participant recruitment were carried out with assistance of bilingual community workers through the MRP and community organisations. To maximise the diversity of the study sample, participants were purposively recruited by country of origin, focusing on countries that have high and moderate collectivistic culture [26, 27]. Based on the Hofstede’s cultural dimension theory and model [26, 28], Table 1 provides the Individualism-collectivism score for each country participating in the research. Table 1 also details the demographic characteristics of focus group participants. Data were collected using community based consultative focus groups discussions (FGDs) from fourteen FGDs with a total of 164 participants. FGDs were based on the principles of Participatory Action Research frameworks [29] and have proven useful to understand various emerging issues with new migrants and changing family dynamics after migration [2, 3]. FGDs centred around involving local migrant community members and representatives in research, knowledge exchange and planning to account for community partnership, shared power, building community capacity, and social change [30, 31].

**Table 1:** Demographic composition of focus group participants.

FGD No	Country of birth	Individualism-Collectivism score+	No. of people	Age range (Years)	Median age (Years)	Gender make up	Migration streams	Length of stay in Australia in Years: Median (range) (in Years)
1	Afghanistan	50	13	21–62	54	100% F	Refugees and family reunion	8 (1–27)
2	Afghanistan	50	10	58–77	66.5	100% M	Refugees/humanitarian entrants and family reunion	19 (6–24)
3	Eritrea, Ghana, Liberia, Sierra Leone, and Sudan	15–20	13	24–58	49	63.6% F; 36.4% M	Refugees/humanitarian entrants and family reunion	10 (0–12)
4	Ghana, Rwanda, and Uganda	15–20	7	31–57	35	14.3% F; 85.7% M	Refugees/humanitarian entrants and family reunion	1 (0–11)
5	Bangladesh	20	13	24–47	40	23.1%F; 76.9% M	Skilled/Family reunion	6 (1–19)
6	Bangladesh	20	11	22–52	40	36.4%F, 63.6%M	Skilled/Family reunion	12 (1–16)
7	Burma	-	13	23–80	38	61.5%F, 38.5%M	Refugees/humanitarian entrants and family reunion	8 (0–30)
8	India	48	17	20–68	38.5	100%F	Refugees/humanitarian entrants and family reunion	3 (1–25)
9	India	48	14	18–65	43	64.3%F; 35.7%M	Skilled/Family reunion	4 (0–7)
10	Iraq	30	11	36–66	52	100%F	Refugees/humanitarian entrants and family reunion	2 (0–5)
11	Iraq	30	10	28–69	55.5	10%F; 90%M	Refugees and family reunion	1.5 (0–4)
12	Iraq	30	6	20–29	23	100%F	Refugees/humanitarian entrants and family reunion	6 (2–8)
13	Nepal	30	15	21–59	47	100%F	Refugees/humanitarian entrants and family reunion	5 (1–6)
14	Nepal	30	11	46–73	59.5	100%M	Refugees/humanitarian entrants and family reunion	2.5 (0–7)

+ based on Hofstede’s cultural dimension theory and model, estimated score range: 5–39: high collectivistic and low individualistic culture; 40–70: moderately collectivistic culture; 71–100: high individualistic and low collectivist culture

Recruiting FGD participants from known networks ensured increased participation and helped manage issues of group dynamics [32]. As most of these groups met regularly, the data collection for the research was not a part of the ‘only-once group’ [33]. The relationships of trust existing within the established networks formed a foundation that enabled the focus group facilitator to push boundaries, explore specific issues and have rich discussions. Prior to data collection, researchers and bilingual community workers met the community members and explained the study’s objective and expected outcomes through a plain language statement. Participants were explained the voluntary nature of participation. Participants were invited to take part in the study to form new groups or as part of existing community groups. The average FGD size was eleven, which is considered within an adequate range of participants in focus groups [34]. However, they were a few focus groups with increased participation which could be due to the collectivist cultural background of participants, as family members and friends groups came together for participation. Written consent was obtained from all participants except for those who had low text based literacy where verbal consent was audio-recorded. Participants were each given AUD $25 supermarket voucher in appreciation of their time. The FGDs were facilitated by a three member team: facilitator, interpreter/bilingual community worker and a note taker, an approach that has been successfully used previously [2, 3, 35, 36]. FGDs were conducted using a standard interview guide. The interview guide was developed in consultation with the MRP. The facilitator’s experience allowed him/her to balance the discussion to include all participants. Interpreters/bilingual community workers translated the questions and also probed, as needed to clarify responses and for better discussion. In order to minimise the interpreters or bilingual community workers influence and bias, at the end of each FGD question, all responses were summarised back to FGD members seeking their affirmation of the accuracy and completeness. The research employed interpreters or bilingual community workers for each of the communities participating in the research, these workers were of similar ethnic background as the group they were supporting and had been working with these communities for many years. The note taker took notes (in English language) of each discussion and also key observations such as participants’ responses, discussions before and after the FGD and any queries for clarifications, which assisted in data analysis. FGDs were conducted in community settings, such as community halls or centres and public libraries, as suited the comfort of participants. Each discussion lasted for around ninety minutes. FGDs were audio recorded and later transcribed verbatim by professional transcribers for analysis. The FGD guide is provided as appendix 1.

### Data analysis

The data transcripts were manually coded independently by one member of the research team (NW) and subsequently verified by the second researcher (AR). Data were coded using the six step process of Braun and Clark: (i) familiarization with the data by reading and re reading the transcripts and field notes; (ii) generating initial codes and inserting the initial codes in the transcripts; (iii) developing and searching for themes and grouping the codes into developing relevant themes; (iv) reviewing the themes against the coded extracts and the data extracts and create a thematic ‘map’; (v) defining and naming the themes and sub themes; and (vi) narrating the themes and sub themes, with a selection of participant voices for each theme [37]. The written notes assisted data coding and final thematic mapping. Discussions were held amongst the authors and MRP to confirm the emerging themes and resolve any differences.

### Findings

Major themes that emerged from analysis were: (i) Changing gender roles and women empowerment; (ii) sending money home; (iii) culture shock and increased intercultural conflict; (iv) change in lifestyle from collective to individual culture; and (v) role of extended community in mitigating culture shock (Table 2).

**Table 2:** Major themes and subthemes from the analysis.

FGD No.	1	2	3	4	5	6	7	8	9	10	11	12	13	14
**Themes and subthemes**														
**Women empowerment and changing gender roles**														
*More right for women by law*	√	√	√	√	X	√	√	X	√	√	√	√	√	√
*Increased labour and burden with the house work and joining the workforce for women*	√	X	X	X	√	√	√	X	√	X	X	X	√	X
*Women joining the workforce and financially independent*	√	√	√	√	X	√	√	X	√	√	√	√	√	X
*Changing of traditional gender roles*	X	√	√	√	√	√	√	X	√	√	√	X	√	X
*Men contributing to household work*	X	√	X	√	√	√	√	X	√	√	√	X	√	X
*Finances linked to power within the family*	√	√	√	√	√	X	√	X	√	√	X	X	√	X
*Men losing their power within the family system*	√	√	√	X	X	√	√	X	√	√	√	X	X	√
**Sending money home**														
*Pressure to send money back home*	√	√	√	X	X	X	X	X	X	X	X	√	X	X
*Family conflict*	√	√	√	X	X	X	X	X	X	X	X	√	X	X
**Culture shock and increased intercultural conflict**														
*Culture shock- pressure to fit in*, *intercultural conflicts within the home and society*	√	√	√	√	√	√	√	√	√	√	√	√	√	√
*Limited social circle due to language and cultural barriers*	X	√	X	√	√	X	√	√	√	√	√	√	√	√
*Loneliness and social isolation*	X	√	X	√	√	X	√	√	√	√	√	√	√	√
**Change in lifestyle from collective to individual cultural values**														
*Lack of family and community support especially for child rearing and teaching children about culture*	X	X	√	√	√	√	√	√	√	√	X	X	√	X
*Changing lifestyle with limited time to communicate with family and socialise*	X	X	√	√	√	√	√	X	X	√	X	√	√	√
*Consistent effort to connect with family and friends- watching videos/news of back home*	X	X	√	√	X	X	√	√	X	√	X	√	X	X
**The role of extended community in mitigating culture shock**														
*Positive experiences of engaging with community groups*	√	√	√	X	√	√	√	X	√	√	√	X	√	√
*Community as the new extended family- support in the new culture*, *protect*, *and preserve culture*	√	√	√	X	√	√	X	X	√	√	√	X	√	√

√ if mentioned, X not mentioned

### Women empowerment and changing gender roles

Female participants across most of the focus groups had feelings of empowerment and freedom being in Australia because they felt less restricted and more independent in the new culture. They were financially independent with the welfare support and had the opportunity to work outside the house. Women especially those from South Asian background noted that although they successfully negotiate this new identity within their families, there were cases where they had to manage working both outside the home and undertaking all of the household work. Female participants pointed that these instances led to exhaustion and tiredness and conflicts in families. As a participant shared her experience:

‘If women have to go to work, when they come back husband will say ‘oh I’m tired I’ve been to work’ so the wife has to leave them for a while watching television and have to work in the kitchen even though she has gone to work, but kitchen work is her work’ (Nepalese female participant, FGD 13)

Most of the male participants shared that they come from a patriarchal background where men have authority over family decision-making and were the ‘head’ of the family. They found the transition into Australian society particularly difficult and felt that the traditional male and female roles within the family were challenged. These changes were described as men’s increased participation in household and child care work with chores such as cooking and changing nappies, that were previously thought to be a woman’s ‘job’ in the family, a participant noted:

‘That was the most difficult thing I have seen here … it was my first time to change the nappy so it was hard for me. It was a good experience but it was also hard for me to digest that one because I was always saying to myself that this is not my job you know’ (African male participant, FGD 4)

Consequently, some male participants said they were enjoying the changed family functioning as it gave them an opportunity to support in their household. A participant recounted his experience:

*‘*It doesn’t matter if my wife is working or not, the kids they are my kids, too, I also need to worry about what’s going on. I love to clean my own house’ (Bangladeshi male participant, FGD 6)

It was observed across most of the focus groups that the ability to contribute money to run the house was associated with power. Male participants particularly found it difficult as their decision making and financial authority was questioned within the family towards a more balanced division of power between couples. This often affected family cohesion and functioning.

‘Back home one person, head of the family; usually the father was working, financially supporting, and then all the issues were resolved, family issues, whatever issues, was resolved by that same person. He decided and we respected the decision of the head of the family, based on a consultation. But here individually everyone works, everyone financially supports themselves, so we feel that individuality has an impact on us. Yeah it was a big shock…’ (Afghani male participant, FGD 2)

### Sending money home

Although participants noted that they were struggling financially, they stressed the need to send money back home to their family and friends who were totally dependent on this money for survival. Sending money home was an additional stressful responsibility and an obligation because participants recalled that their family had supported them while they needed it the most. Their families were struggling to make ends meet either at a refugee camp or in a conflict zone. This burden was shared commonly amongst the Afghani, African and Iraqi groups coming from humanitarian backgrounds. The pressure to send money often resulted in conflict between couples as each prioritising sending money to their own family over their partner’s family. As a female Afghani community bilingual worker highlighted,

‘Most of the time conflicts between husband and wife are caused by sending money back home’ (Afghani bilingual worker, community organisation)

### Culture shock and increased intercultural conflict

Participants although respecting the new host culture/s often felt a stark difference compared to their background culture. All participants increasingly felt the pressure to adapt and ‘fit in’ the new culture. Participants shared apprehensions of the new ways of dressing and even the way of talking, for example a youth female participant shared she struggled looking in the eye while communicating.

‘I had to speak the last because I was a female, but here in Australia they giving you your right to speak all the time, speak up and …then they tried to train me how to speak, how to look to the eyes of people, 'cause it's like respecting them’ (Iraqi female participant, FGD 12)

Majority of participants shared that with limited familiarity socially along with language and cultural barrier they were not confident and comfortable interacting with the wider community and often struggled with loneliness. As a female participant asserted:

‘Isolation is the main problem. If you are not working then you are totally isolated, if you don’t have a very big support of your relatives, friends and family then you are totally isolated. So either you have your relatives, friends, big circle or you are working otherwise you are just isolated’(Indian female participant, FGD 8)

The increased intercultural conflict along with the lack of traditional social and community structures exacerbated their acculturation stress, as they struggled to cope in the new culture and environment.

### Change in lifestyle from collective to individual cultural values

Participants recalled their home culture as a collective culture of interdependence with a lot emphasis on role of family, friends, neighbours and community, which influences day to day decision making and the culture of doing things together. Additionally, they served as a source of support and an enabling factor to cope with challenges and stresses in life. This finding was common across most groups except the Afghani, Iraqi and Nepalese men’s group.

Migrant families conceptualised family as capital calling it as their “riches” and “asset” but noted a loss of this capital after migration characterised by impaired family unit and cohesion. In addition, post migration society is driven by individual cultural values and changed lifestyles of more independence and individual living. As a participant shared her experience:

‘I think the individual thing here … is a big negative for us in many ways because it just divides us. We are separated. It is separating us’ (African female participant, FGD 3)

Most female participants especially felt an increased pressure in the individualistic culture, with a background of sharing work and doing things together they felt a deep absence of family support and lack of communal spaces that would facilitate such gatherings. These traditional forms of support not only helped them share the physical labour but also served as a platform for social interactions with friends and family ensuring their emotional well-being.

‘For women it’s hard to manage everything so if someone works, wants to earn money and bring home the money and then she has to manage everything around the house. Back home there were grandparents, aunties, uncles and they were all around the corner which wasn’t too far’ (Burmese female participant, FGD 7)

Participants pointed out that in their home country they could visit their friends and families at any given time without necessarily planning it. Whereas, now with the changed lifestyle they were busier than before with English language classes, JobActive or household work and had to always plan ahead. A participant pointed out:

‘There is no social life. There is no family. You can't find enough time to talk to your own children or to share their life’ (Iraqi female participant, FGD 10)

Some participants, mainly from African, Burmese, Indian and Iraqi groups, shared that they missed their home culture and people and thus made consistent efforts to connect with their families and friends back home. They also kept themselves updated with the news of their home country while not being as keen to explore their present environment.

### The role of extended community in mitigating culture shock

Participants shared a common joy of meeting members from their country of origin or with similar ethnic background, be it at community events, social groups, at a park or otherwise socially. These community members helped them feel comfortable and filled the void of missing family and friends. With similar cultural beliefs and background participants related to the community members as their extended families and close friends. This finding was more common amongst the middle-aged and older participants as compared to the younger participants. These participants felt more isolated due to the language barrier and culture differences. As a community bilingual worker points out of his experience working with the community members:

‘I have like nearly 50 or 55 clients, all of them when arrive they come to [organisation]. . . .at that time they didn't know anyone … And nowadays become very close friends, it [community social group] has become one family. And they wait every Friday for social group, now we share their happiness’ (Iraqi community bilingual worker, community organisation)

Participants shared that they were also supportive and protective for their community members, as shared:

‘Now the children or you know the community interact, so the children would know–adults, when they see adults around … It’s our duty. One time right in the shopping centre, I was coming out from the shop, and I saw one of my community’s son standing with the police. I was sure they pass by my business. No, it’s my business … So it must be our duty as an African to protect our children. Don’t say, because the child is not my child’ (African female participant, FGD 3)

Participants felt comfortable being closely linked with their community members and sharing their problems and often identified common issues that helped ease their stress. They felt comfortable as they shared common cultural values and beliefs which helped calm their fear of losing their culture. These community groups also served as platforms to celebrate their festivals and cultures together.

## Discussion

This study explored how migrant families coming from collective cultures cope with social and cultural changes while settling in Australia. It sought to understand how the absence of traditional forms of support affects their daily lives and family functioning and the enhanced role of local community, people with similar ethnic and culture background, in the settlement experiences of migrants and their families.

The study participants associated the shift from collectivistic and patriarchal society to an individualistic and egalitarian society to be challenging and difficult. The absence of collective spaces, familial and communal support as participants struggled with acculturation and the changing family dynamics further added to their acculturative stress, negatively impacting their settlement journey. These findings concur with existing research of migrant families’ experiences upon migration to Australia [2, 3, 12, 15, 35, 38]. Renzaho et al (2010–11) highlight struggles of migrant families with acculturation, changing family functioning and power dynamics upon migration and its effect on family cohesion and unity. Our study findings suggest increase in conflicts upon migration within families and between couples due to the changes in family functioning and disruption in the traditional gender roles, which is consistent with existing literature on differences in acculturation stress experienced by couples [10, 39, 40]. Another aspect although not extensively covered in this scope of this study was the stress and family conflict associated with financial issues and sending money back home to friends and family, which is has been identified as a major cause of conflict within migrant families in research [10].

In addition, our research highlights an important issue that migrants face in their settlement journey, which is the absence of traditional forms of social support for daily functioning and social engagement. These traditional forms of support were identified as an enabling factor that helped deal with stressful situations at a family, community and society level. Upon migration an absence of this support exacerbates the existing acculturative stress affecting the migrants’ well-being and negatively impacting their ability to cope with change. Participants, more commonly women, expressed feeling confused, lost, and isolated with the lack of family and social support for household chores and childcare work. Participants also identified language as a barrier in interacting and engaging with the local people, new system and culture. Babatunde-Sowole et al. (2016) point out experiences of African female migrants to cope with household work and working for an income in the absence of communal and familial assistance often led to physical exhaustion and further affecting their mental well-being. Research suggests that migrants feel isolated in their host country and experience issues such as language barrier, finding suitable employment [41, 42] and acculturative stress while adjusting to the new environment, all of which can have psychological impact and affect their well-being [10, 43, 44]. Research asserts the importance creating a milieu with optimal social capital and relationships for positive migration experiences [12, 45].

Our study found that participants although felt lonely and isolated they tried to make conversations and reach out to people with similar cultural backgrounds and languages, which could be also attributed to their collectivist background. They ensured to join community social groups, as it helped them form bonds with people coming from similar cultures and languages and facing similar issues that helped ease their stress and anxiety [46]. Participants relied on these forms of social capital in the form of social ties embedded in the ethnic community as a substitute for social support otherwise provided by their families and close friends. This source of support, familiarity and comfort, positively influenced their settlement and integration experience upon migration. Social capital bonding was more pronounced in our research which could be due to the language and cultural barriers participants experience upon migration; Social capital bridging was also observed with participants from similar cultures and situations. Presently there is limited literature on the changing forms of social capital and how it influences the settlement and integration journey of new migrants within Australia. It will also be beneficial to understand the impact of lack of traditional forms of support on the general well-being of individuals and families upon migration to Australia.

### Limitations, policy implications, and conclusion

The study has some limitations. FGD participants were recruited from established networks of existing community groups and interpersonal relations, limiting the diversity within each group, and hence affecting the representativeness of the sample. Due to the exploratory nature of the study, research was conducted with migrants from mixed backgrounds of skilled and family reunion, and humanitarian streams. It is useful to highlight that our research emphasizes on social capital bonding that can have negative consequences as well, with limited or no communication with wider community and being limited to one’s own comfort spatially-socially and being over protected with one’s own culture and people [22]. Further close engagement and research may be required to replicate, test or confirm these findings in wider migrant communities of the Western Sydney region.

Notwithstanding these limitations, the findings point to the need for policies and settlement services to be culturally competent and reflect an awareness of the cultural changes from a collective context to a Western individualistic culture. Factors such as acculturative stress and absence of social capital that lead to social isolation and loneliness amongst migrants need to be acknowledged in policies and settlement services. More interventions are required to focus on helping migrants cope better with these life challenges. This study suggests sensitivity is required to support migrants and migrant families as they embark on their new life in Australia. Our findings suggest an increased burden on female migrants. Recently there have been increased discussions in Australia for improved family friendly work practices that encourage family-life balance, flexible working hours and provision of child care. However, these debates remain limited to the general Australian population excluding migrant women, and are also limited to public spaces and exclude unpaid work done at home and/or family responsibilities [47, 48]. These debates and discussions need to be widened to include the specific needs of migrant women that consider issues of changing family dynamics, conflicts and gender and to influence program design. Our research also found that male migrants experience a loss of power and authority within a family upon migration. The present service provision in Australia does not cater to the specific needs of migrant men and all services are clubbed under the umbrella of family services. It will be useful to have culturally sensitive services such as support groups for migrant men. At present, community organisations and Migrant Resource Centres in Australia focus on providing settlement and integration services by organising regular social groups or support groups [49–51]. As our research participants particularly highlight the importance of community groups in mitigating the culture shock upon migration, it will be useful to adopt a social capital framework which lends support to the development of community interventions that addresses ways in which cultural and social networks operate and communication occurs within families and extended families across cultures, especially in the absence of wider family networks. Further research on the changing social capital upon migration and its influence on the settlement journey of migrants will be useful. Research on pre-migrant experiences and collective cultural coping strategies as migrant families adapt to the new environment will also add value to existing literature and inform policy.

## Appendix 1. Focus group discussion guide

Introduction and warm up: demographic data

1. Can you tell us about your experiences of moving to Australia? (Probe: what has been your experience in the post migration phase?)
2. Have you experienced any intercultural conflicts and/or culture shocks?
3. To what extent has migrating to Australia led to any changes in the family dynamics? (Probe: changes to family structure and roles, family support structure, or social circle and friends? How does it differ from back home?)
4. How do you interact or connect with community support services in Australia? (Probe: Which ones? Do they benefit you? In what way?)
